# Exploring the relationship between dissociative experiences and recovery in psychosis: cross-sectional study

**DOI:** 10.1192/bjb.2024.113

**Published:** 2026-02

**Authors:** Claudia Calciu, Rob Macpherson, Kerry J. Rees, Sui Yung Chen, Sarah Ruxton, Rhiannon White, Mazen Almaskati, Francesca Hill, Anca Vasilis-Peter, Sebastian Desando, Oliver Pennell, Carolyn Nasubuga, Jackie Webb, Mark Walker, Camelia Soponaru

**Affiliations:** 1 Gloucestershire Health and Care NHS Foundation Trust, Gloucester, UK; 2 ‘Alexandru Ioan Cuza’ University, Iasi, Romania; 3 University of Gloucestershire, Cheltenham, UK

**Keywords:** Dissociation, psychosis, recovery, compartmentalisation, detachment, absorption

## Abstract

**Aims and method:**

This study explored the association among dissociative experiences, recovery from psychosis and a range of factors relevant to psychosis and analysed whether dissociative experiences (compartmentalisation, detachment and absorption) could be used to predict specific stages of recovery. A cross-sectional design was used, and 75 individuals with psychosis were recruited from the recovery services of the Gloucestershire Health and Care NHS Foundation Trust. Five questionnaires were used – the Dissociative Experiences Scale – II (DES), Detachment and Compartmentalisation Inventory (DCI), Questionnaire about the Process of Recovery, Stages of Recovery Instrument (STORI), and Positive and Negative Syndrome Scale – and a proforma was used to collect demographic data.

**Results:**

Our findings indicated that compartmentalisation, detachment and absorption, as measured by DES and DCI, do not predict stages of recovery as measured by the STORI.

**Clinical implications:**

The results of this study suggest that there is no simple relationship between dissociative and psychotic symptoms. They also suggest a need to assess these symptoms separately in practice and indicate that special approaches to treatment of psychosis may be needed in cases where such symptoms have a significant role.

Psychosis is a highly disabling illness that affects those suffering from it, their families and the entire society. This illness has a 15–20-year mortality gap compared with the general population, and it has significant human and financial costs. Andrew et al^1^ estimated a total societal cost in England of £11.8 billion per year, and a cost to the public sector of £7.2 billion. A study commissioned by Rethink estimated that the health service spent £2.0 billion on services for people with psychosis in 2012–2013.^2^ This demonstrates that recovery-focused interventions are clinically and financially effective and could contribute to savings to be reinvested in care.

Our study investigated how psychosis, in its recovery phase, could be related to dissociation. An overview of the literature was conducted^3^ before the development of the current study; this synthesised available data about the relationship between dissociation in psychosis and recovery from psychosis. We found a dearth of well-designed, adequately powered studies; therefore, this matter remains relatively under-researched at this time. These findings and our clinical observations formed the basis for the development of the current study. This study, which is pioneering in its nature, investigated whether there was an association between dissociative phenomena and recovery in psychosis. Our findings pave the way for specific research on this subject.

## Concepts relevant to the study

The study was developed around three concepts: dissociation, psychosis and recovery. We present below the conceptualisations used for the purposes of this study.

### Psychosis

Psychosis and its relationships with other psychopathological phenomena have preoccupied researchers and clinicians for decades. Sadock and Sadock^4^ defined the concept of psychosis as a group of mental illnesses in which loss of reality testing and the boundaries of the self are the main characteristics. The DSM-5^5^ and ICD-10^6^ describe specific diagnostic criteria for different psychotic conditions, of which schizophrenia is the main representative. Owing to severe impairment of the ability to test reality, the psychotic person incorrectly assesses the accuracy of their perceptions and thoughts and makes incorrect inferences about external reality, even in the face of evidence to the contrary. Some of the main psychotic experiences include perceptual abnormalities, thought disorder, disorganised behaviour, cognitive decline, flattening of affect, and impaired motivation and volition. Sadock and Sadock^4^ argue that in mainstream psychiatric practice, the term psychotic has become synonymous with severe impairment of social and personal functioning, characterised by social withdrawal and the inability to fulfil usual roles in daily and occupational life.

### Dissociation

Studies have suggested that dissociation may have a causal role in the development of psychotic symptoms.^7^ In a non-pathological approach, Watkins and Watkins^8^ described dissociation as a ‘natural strategy’ that allows people to adapt. Although there continues to be controversy around the conceptualisation of dissociation,^9,10^ there is increasing evidence that dissociative experiences exist transdiagnostically^11^ and on a ‘spectrum’ from normal to pathological.^12,13^

Dissociation is ‘the fragmentation of the usual continuity of subjective experience’^14^ (p. 265); ‘disruption of and/or discontinuity in the normal integration of conscience, memory, identity, emotion, perception, body representation, motor control and behaviour’, and ‘partial or total loss of the normal integration between the memory of the past, identity awareness and body movements control’.^6^

Different types of dissociative phenomena have been described and explored, for example, identity alteration and amnesia in dissociative disorders^15^ and depersonalisation and/or derealisation in post-traumatic stress disorder and dissociative conditions.^16,17^ Dissociation is now seen as a multifaceted psychological construct,^18^ incorporating a diversity of symptoms and manifestations.^10^ Briere, Weathers and Runtz^19^ (p. 222) described dissociation as a ‘multidimensional construct’.

Holmes et al^10^ and Brown^9^ argued for the existence of two distinct forms of dissociation (detachment and compartmentalisation) with different definitions, mechanisms and treatment implications. Detachment from self and environment is expressed through symptoms such as depersonalisation, derealisation, emotional bluntness, *deja vu* and out-of-body experiences. These are typically caused by life-threatening events and strong emotions.^16^ Detachment was defined by Nijenhuis and Van der Hart as ‘an altered state of consciousness characterised by a sense of separation (or ‘detachment’) from aspects of everyday experience’^20^ (p. 434). Compartmentalisation of normally integrated mental functions such as memory, identity, emotion, perception, body representation and control of voluntary movements is manifested through symptoms including dissociative amnesia, impaired emotional control and impaired identity control.^20^ Brown's description of compartmentalisation phenomena placed them on a ‘continuum of distress and disability, ranging from non-pathological experiences produced using hypnotic suggestion, through milder pathological states such as transient amnesias and conversion disorders, to chronic and extremely disabling conditions like somatization disorder and DID [dissociative identity disorder]’^9^ (p. 14).

Butler^21,22^ introduced the idea of ‘normal’ dissociation, for example, absorption in leisure activities or work tasks. This represents the involuntary tendency to narrow attention, to the extent of ignoring the environment,^23^ and implies a temporary suspension of reflective consciousness.^22^

Dissociative and psychotic experiences can be induced by illicit substances, and the dissociative experience has traditionally been considered a response to psychological trauma.^24^ Therefore, it is important to assess the presence of trauma and substance misuse to provide a more complete understanding of the occurrence of dissociative experiences during recovery from psychosis.

Studies have shown an overlap between psychotic symptoms and dissociative experiences.^16,19^ A recent cross-sectional study by Fung et al^25^ that used network analysis to explore the associations among different psychotic and post-traumatic dissociative symptom clusters confirmed previous findings that post-traumatic stress disorder and dissociative symptoms are closely associated with psychotic symptoms. The suggestion that dissociation can mediate the relationship between trauma and psychosis has been demonstrated by previous studies. Fung et al also showed significant associations of childhood and adulthood trauma with perceptual abnormalities.^25^ The authors emphasised the importance of screening for trauma and dissociation when working with people with psychosis or at risk of developing psychosis.

Research on the aetiology of dissociation has investigated the brain activity that occurs during dissociative experiences. A functional neuroimaging script-driven imagery study conducted by Mertens et al^26^ explored the neural correlates of acute post-traumatic dissociation, finding enhanced activation in the cerebellum, occipital gyri, supramarginal gyrus and amygdala during trauma recall. Further pioneering research by Schäflein and colleagues^27^ identified behavioural correlates of acute dissociation by measuring frontal electroencephalography in people with dissociative disorders, following a stress-inducing facial mirror confrontation paradigm. Their findings suggested the possibility of altered neural processing in this group of dissociative patients. More research is needed to explore brain activity during dissociative experiences; this would contribute to a more in-depth understanding of dissociation as a complex phenomenon and would refine the clinical repertoire of therapeutic interventions.

Although psychosis and dissociation can be argued to be distinct, ‘sense of agency’ seems to be common to the complex mechanisms governing the phenomenology of psychosis and dissociation. It has been studied in relation to both groups of phenomena. Here, ‘sense of agency’ refers to the experience of initiating and controlling an action.^28^ Studies have so far focused on the neurobiological basis of psychotic symptoms, highlighting the role of structural and functional brain abnormalities and neurotransmitter system dysfunction.^29,30^ Hallucinations and delusions of control have at their core an atypical perception of agency,^31^ and experiencing oneself as being the source of one's own motor actions is central to self-consciousness.^32,33^ It has been proposed that problems with sensorimotor predictions, which are essential for a sense of agency, may lead to action awareness abnormalities in psychosis.^34^ Moreover, in some dissociative experiences, owing to an impaired sense of agency, there is an impaired sense of volition before movement and/or action (for example, dissociative seizures)^35^ and delayed awareness of the intention to move (for example, functional neurological disorder).^36^

### Recovery

The concept of recovery is crucial to the understanding and treatment of psychosis. In the present study, we used Anthony's definition of recovery: ‘a deeply personal, unique process of changing one's attitudes, values, feelings, goals, skills and roles. It is a way of living a satisfying, hopeful, and contributing life even with limitations caused by the illness. Recovery involves the development of new meaning and purpose in one's life as one grows beyond the catastrophic effects of mental illness’^37^ (p. 527).

Although it was previously thought that psychosis invariably had a poor outcome, research^38^ shows that the majority of patients recover from this illness. Recently, researchers have also identified the following stages of recovery,^39^ thereby facilitating understanding of the processes required for patients to re-enter fully into society after a serious illness:

- moratorium – denial, confusion, hopelessness, deprived sense of one's life, loss of purpose in life, self-protective withdrawal;
- awareness – the advent of hope and a sense of personal agency for taking responsibility for recovery and purpose in life;
- preparation – setting new goals;
- rebuilding – active pursuit of personal goals, building a more positive sense of self;
- growth – hopefulness, a positive overlook towards the future.

Our clinical observations from working with patients recovering from an episode of psychosis are that people can live with psychotic symptoms and have a meaningful life, suggesting that they have mental systems that help them manage different ‘compartments’ of their mental life separately. It is unclear how such experiences might become ‘consciously learned and applied’ as psychotherapeutic coping strategies.^18^

## Aims

The aim of the present study was to explore the associations between dissociative experiences and recovery from psychosis and to analyse whether dissociative experiences (compartmentalisation, detachment and absorption) could predict specific stages of recovery.

## Method

### Recruitment

The authors assert that all procedures contributing to this work comply with the ethical standards of the relevant national and institutional committees on human experimentation and with the Helsinki Declaration of 1975, as revised in 2008. All procedures were approved by the Health Research Authority Health and Care Research Wales Committee (reference number: 20/EM/0218).

Seventy-five individuals with psychosis were recruited from the recovery services of the Gloucestershire Health and Care NHS Foundation Trust. The research was publicised by flyers and presentations to adult mental health community teams and the two recovery units in the trust. In all teams, participants were identified by screening the case-load for patients with a diagnosis of psychosis; this was done by consultant medical staff working in the clinical team or in consultation with clinicians (medics or care coordinators). The database held by the research department in the trust, which includes information for patients who have agreed to be contacted regarding research projects, was also screened. Individual patients indicated interest by communicating with their care coordinators or their allocated psychiatrist. When individuals expressed an interest in participating, a convenient time for participation was arranged. Following standardised training, researchers comprising clinicians and psychiatry trainees collected data in face-to-face sessions or using phone calls.

The inclusion criteria were as follows:

- diagnosis of psychotic disorder (first episode or chronic illness);
- age between 18 and 65 years;
- any gender.

Participants were required to have been diagnosed with one of the psychotic illnesses listed below, with diagnoses made on the basis of the ICD-10 criteria:^6^

- F20 schizophrenia (any type);
- F25 schizoaffective disorder;
- F22 delusional disorder;
- F29 psychosis not otherwise specified;
- F53.1 puerperal psychosis;
- F23 brief psychotic disorder.

The exclusion criteria were as follows:

- patients who lacked capacity to consent to participate;
- patients with acute psychotic illness and as a result considered by their team to be too unwell to participate.

Written consent for participation, use of data provided and dissemination of findings was obtained from all participants.

### Design

Using a cross-sectional design, we explored predictors of recovery from psychosis with a focus on dissociative experiences (compartmentalisation, detachment and absorption) in a clinical population diagnosed with psychosis.

## Instruments

The following measures were used in this study.

### Instruments measuring dissociative experiences

#### The Detachment and Compartmentalisation Inventory (DCI)^40^

This scale contains 22 items corresponding to two subscales: ten items assessing compartmentalisation, ten items assessing detachment and two items examining valid responding. The answers are scored on an eight-point scale (0 indicates that an experience described by an item ‘never’ occurs, and 7 indicates a daily frequency). A higher score indicates increased experiences of detachment and compartmentalisation. Internal consistency was suitable; Cronbach's alpha = 0.895.

#### The Dissociative Experiences Scale – II (DES-II)^41^

This scale contains 28 questions designed to measure dissociation in clinical and non-clinical populations. It includes three subscales: amnesia, absorption and detachment. The items are answered on an 11-point percentage scale, which measures the frequency with which each of the experiences described in the items occurs in daily life (0% means that an experience described has never happened, and 100% means that it occurs very frequently). A higher score relates to frequent dissociative experiences. Internal consistency could not be calculated owing to a lack of scores greater than zero.

### Instruments measuring recovery from psychosis (recovery, stages of recovery)

#### The Stages of Recovery Instrument (STORI)^42^

This measure assesses a participant's definition of recovery and contains 50 items organised in ten groups of five items, where each item in a group corresponds to a stage of recovery. The items are answered on a six-point scale (0 indicates ‘Not true at all now’, and 5 indicates ‘Completely true now’). The participant's scores are calculated for each stage, and they are then allocated to the stage with the highest score. If scores in different stages are the same, the participant is allocated to the highest stage. A higher stage indicates a more advanced stage of recovery. Internal consistency was suitable; Cronbach's alpha = 0.899.

#### The Questionnaire about the Process of Recovery (QPR)^43^

The QPR includes 15 items designed to assess aspects of recovery that are meaningful to patients. Its scores are strongly associated with general psychological well-being, quality of life and empowerment, all of which are crucial in recovery from psychosis.^29^ The items are scored on a five-point scale, from 0, ‘Disagree strongly’, to 4, ‘Agree strongly’. A higher score indicates a more advanced stage of recovery. Internal consistency was suitable; Cronbach's alpha = 0.923.

### Instruments measuring symptoms of psychosis

#### The Positive and Negative Syndrome Scale (PANSS)^44^

PANSS is a semi-structured interview that contains 30 items corresponding to three subscales for: positive symptoms (delusions, grandiosity, suspiciousness/persecution, unusual thought content); negative symptoms (blunted affect, emotional withdrawal, poor rapport, passive/apathetic social withdrawal, lack of spontaneity and flow of conversation, active social avoidance); and general psychopathology (e.g. anxiety, low mood, elevated mood). The answers are scored on a seven-point scale (1 indicates ‘absent’, and 7 indicates ‘extreme’). A higher score indicates a higher level of psychotic symptoms. Internal consistency was suitable, with Cronbach's alpha = 0.898.

### Other instruments

A proforma was used for information related to demographics, illness and social background.

## Results

There were comparatively few instances of missing data, and any missing data were not transformed. The mean age of participants was 39.65 years, and 65.3% were female. The majority of the participants (46.7%) had a diagnosis of schizophrenia. Only 5.3% had a history of significant substance misuse. All other variables are represented in Table 1.

**Table 1:** Demographics

	mean	s.d.
Duration of illness, months	134.67	122.77
Time since last episode, months	37.77	71.39
	*N*	%
**Ethnicity**		
White	62	82.7
White and Black Caribbean	3	4.0
Indian	3	4.0
Other	7	9.3
**Marital status**		
Married	11	14.7
Unmarried but in a relationship	6	8.0
Single	58	77.3
**Education**		
Primary	22	29.3
Secondary	51	68.0
FE/A levels	2	2.7
**Diagnosis**		
Schizophrenia	35	46.7
Schizoaffective	13	17.3
Delusional disorder	3	4.0
Psychosis – not specified	15	20.0
Puerperal psychosis	1	1.3
Brief psychotic disorder	8	10.7
**History of trauma**		
No issues	45	60.0
Significant trauma	30	40.0
**Current medication**		
Taking medication	73	97.3
Not taking medication	2	2.7
**Psychotherapy or interventions**		
CBT	27	36.0
Psychoanalytical	3	4.0
Other type	6	8.0
None	39	52.0
**Community team**		
GP	4	5.3
Recovery	34	45.3
GRIP	23	30.7
AOT	14	18.7
**Substance misuse**		
No issues	58	77.3
Occasional use	13	17.3
Heavy use	4	5.3
**Employment**		
Unemployed	51	68.0
Voluntary work and/or training	5	6.7
Employed	18	24.0
Retired	1	1.3
**Data collection**		
Face to face	49	65.3
Telephone	26	34.7

AOT, Assertive Outreach Team; CBT, cognitive–behavioural therapy; FE, further education; GRIP, Gloucestershire Recovery in Psychosis (Early Intervention in Psychosis); GP, general practitioner.

The results from the main rating scales, as means and standard deviations, are reported in Table 2. Table 3 shows how many participants were identified as being in each of the recovery stages as per the model described by Andresen et al.^39^ The majority (26.7%) were assessed as being in the third stage of the recovery process, with equal percentages (22.7%) of participants in stage 1 and stage 4. The smallest percentage were placed in stage 2 (9.3%).

**Table 2:** Rating scales scores

	mean	s.d.
DES	175.95	132.16
DCI	47.49	25.48
QPR	64.49	13.32
PANSS	21.60	5.22

DCI, Detachment and Compartmentalisation Inventory; DES, Dissociative Experiences Scale; PANSS, Positive and Negative Syndrome Scale; QPR, Questionnaire about the Process of Recovery.

**Table 3:** STORI classification

Stage	*N*	%
1	17	22.7
2	7	9.3
3	20	26.7
4	17	22.7
5	14	18.7

STORI, Stages of Recovery Instrument.

### Ordinal regression model

An ordinal regression analysis was used to investigate history of substance misuse and history of trauma as categorical predictors and DES, DCI, QPR and PANSS as continuous predictors of STORI. The results are shown in Table 4. The model was assessed for assumptions *a priori*: goodness of fit was supported (*P* = 0.19); test of parallel lines was supported (*P* < 0.001); effect size, Nagelkerke = 0.143; the model accounted for 14.3% of variance.

**Table 4:** Scores within the regression model and/or levels of predictiveness

Predictor	Unit change	Odds ratio	CI	s.e.	Wald	Significance
QPR	0.41	1.042	1.003–1.082	0.019	4.554	0.033
PANSS	−0.084	0.920	0.840–1.007	0.046	3.301	0.069
History of substance misuse (no issues)	−0.516	0.597	0.084–4.243	1.00	0.266	0.606
History of substance misuse (occasional use)	0.543	1.721	0.182–16.305	1.147	0.224	0.636
History of trauma	−0.499	0.607	0.241–1.527	0.471	1.124	0.289
DES	0.000	1.000	0.996–1.005	0.002	0.041	0.840
DCI	0.014	1.014	0.990–1.039	0.012	1.291	0.256

DCI, Detachment and Compartmentalisation Inventory; DES, Dissociative Experiences Scale; PANSS, Positive and Negative Syndrome Scale; QPR, Questionnaire about the Process of Recovery.

QPR was a significant predictor. A one-unit increase in QPR was associated with an expected 0.041 increase in ordered odds of being in a higher level of STORI, with an odds ratio of 1.042 (CI = 1.003–1.082; s.e. = 0.019, Wald = 4.554, *P* = 0.033).

PANSS was a marginally significant predictor. A one-unit decrease in PANSS score was associated with a 0.084 increase in ordered odds of being in a higher level of STORI, with an odds ratio of 0.920 (CI = 0.840–1.007; s.e. = 0.046, Wald = 3.301, *P* = 0.069). In other words, as the PANSS score (psychotic symptoms) decreased, there was a higher probability of being in a higher stage of recovery, although this was not a strictly significant result.

All other predictors were non-significant.

### Correlations

We conducted a correlation analysis to obtain more information about how the two concepts of dissociation and psychosis could be related. The results are presented in Table 5. We found that PANSS was not correlated with other measures; QPR was significantly negatively correlated with DCI (*r* = −0.45, *P* < 0.001) and DES (*r* = −0.36, *P* = 0.002); and DCI and DES were positively correlated (*r* = 0.66, *P* < 0.001).

**Table 5:** Correlation table and descriptive data regarding the interaction effect between variables

Variable	*N*	mean	s.d.	1	2	3	4	5	6
1 (duration, months)	75	134.67	122.77	–					
2 (time since last episode, months)	75	37.77	71.39	0.42**	–				
3 (DES)	75	175.95	132.16	0.15	0.39	–			
4 (DCI)	75	47.49	25.48	0.16	0.10	0.66**	–		
5 (QPR)	75	64.49	13.32	−0.26*	0.18	−0.36**	−0.45**	–	
6 (PANSS)	75	2.16	5.27	0.09	−0.06	0.16	0.20	0.03	–

DCI, Detachment and Compartmentalisation Inventory; DES, Dissociative Experiences Scale; PANSS, Positive and Negative Syndrome Scale; QPR, Questionnaire about the Process of Recovery.

*P < 0.05, **P < 0.01.

To further investigate the unexpected lack of association between QPR and STORI and to identify potential associations among the categorical variables, history of trauma and history of substance misuse, and other variables, Spearman's correlations were calculated (Table 6). The results showed that STORI was not correlated with any other variable. Conversely, DCI and DES were significantly negatively correlated with QPR. In other words, the more advanced one is in the recovery process, the less dissociative mechanisms are used.

**Table 6:** Spearman's correlation table and descriptive data

			History of trauma	Substance misuse	STORI stage incardation	QPR total score	PANSS composite scale	DCI total score	DES total score
Kendall's tau-b	History of trauma	Correlation coefficient	1.000	0.049	0.080	−0.168	0.057	−0.058	0.007
		Significance (two-tailed)		0.667	0.444	0.082	0.567	0.545	0.944
		*N*	75	75	75	75	75	75	75
	Substance misuse	Correlation coefficient	0.049	1.000	0.142	−0.043	0.161	0.151	0.198*
		Significance (two-tailed)	0.667		0.167	0.651	0.098	0.109	0.035
		*N*	75	75	75	75	75	75	75
	STORI stage incardation	Correlation coefficient	0.080	0.142	1.000	0.100	−0.080	0.032	0.011
		Significance (two-tailed)	0.444	0.167		0.249	0.371	0.710	0.903
		*N*	75	75	75	75	75	75	75
	QPR total score	Correlation coefficient	−0.168	−0.043	0.100	1.000	−0.032	−0.290**	−0.225**
		Significance (two-tailed)	0.082	0.651	0.249		0.695	<0.001	0.005
		*N*	75	75	75	75	75	75	75
	PANSS composite scale	Correlation coefficient	0.057	0.161	−0.80	−0.032	1.000.	214**	0.213**
		Significance (two-tailed)	0.567	0.098	0.371	0.695		0.009	0.010
		*N*	75	75	75	75	75	75	75
	DCI total score	Correlation coefficient	−0.058	0.151	0.032	−0.290**	0.214**	1.000	0.517**
		Significance (two-tailed)	0.545	0.109	0.710	<0.001	0.009		<0.001
		*N*	75	75	75	75	75	75	75
	DES total score	Correlation coefficient	0.007	0.198*	0.011	−0.225**	0.213**	0.517**	1.000
		Significance (two-tailed)	0.944	0.035	0.903	0.005	0.010	<0.001	
		*N*	75	75	75	75	75	75	75
Spearman's rho	History of trauma	Correlation coefficient	1.000	0.050	0.089	−0.202	0.067	−0.070	0.008
		Significance (two-tailed)		0.670	0.448	0.082	0.571	0.548	0.945
		*N*	75	75	75	75	75	75	75
	Substance misuse	Correlation coefficient	0.050	1.000	0.161	−0.057	0.192	0.184	0.249*
		Significance (two-tailed)	0.670		0.167	0.629	0.100	0.115	0.031
		*N*	75	75	75	75	75	75	75
	STORI stage incardation	Correlation coefficient	0.089	0.161	1.000	0.146	−0.098	0.039	0.013
		Significance (two-tailed)	0.448	0.167		0.212	0.403	0.737	0.912
		*N*	75	75	75	75	75	75	75
	QPR total score	Correlation coefficient	−0.202	−0.057	0.146	1.000	−0.042	−0.405**	−0.318**
		Significance (two-tailed)	0.082	0.629	0.212		0.718	<0.001	0.005
		*N*	75	75	75	75	75	75	75
	PANSS composite scale	Correlation coefficient	0.067	0.192	−0.098	−0.042	1.000	0.269*	0.294*
		Significance (two-tailed)	0.571	0.100	0.403	0.718		0.020	0.010
		*N*	75	75	75	75	75	75	75
	DCI total score	Correlation coefficient	−0.070	0.184	0.039	−0.405**	0.269*	1.000	0.700**
		Significance (two-tailed)	0.548	0.115	0.737	<0.001	0.020		<0.001
		*N*	75	75	75	75	75	75	75
	DES total score	Correlation coefficient	0.008	0.249*	0.013	−0.318**	0.294*	0.700**	1.000
		Significance (two-tailed)	0.945	0.031	0.912	0.005	0.010	<0.001	
		*N*	75	75	75	75	75	75	75

DCI, Detachment and Compartmentalisation Inventory; DES, Dissociative Experiences Scale; PANSS, Positive and Negative Syndrome Scale; QPR, Questionnaire about the Process of Recovery; STORI, Stages of Recovery Instrument.

* Correlation was significant at the 0.05 level (two-tailed).

** Correlation was significant at the 0.01 level (two-tailed).

## Discussion

We found high levels of dissociative symptoms as measured by DES (*M* = 175.95, s.d. = 132.16) and DCI (*M* = 47.49, s.d. = 25.48) in a group of patients recovering from psychosis. This seems important, as the conventional approaches used to treat psychosis, such as antipsychotics and cognitive–behavioural therapy, are unlikely to have an impact on dissociative symptoms.

Our findings indicated that compartmentalisation, detachment and absorption, as measured by DES and DCI, do not predict stages of recovery as measured by STORI. This suggests that there is no simple relationship between dissociative and psychotic symptoms; for example, that dissociation is one expression of psychotic disease, or that dissociation occurs in more severe psychosis. It also suggests a need to assess these symptoms separately in practice, and that special approaches to treatment of psychosis may be needed in cases where they play a significant part.

It is important to treat the findings of this study with caution. Although the results were accurate with respect to the design we developed, there may be different ways to investigate the relationship between dissociation and recovery in psychosis. Our study was cross-sectional, and it would be helpful to assess individual patients at different stages of their psychosis over time, to understand how dissociation can affect people through their illness history.

DCI was not a significant predictor. We used DCI with the aim of identifying compartmentalisation and detachment-type dissociation, but these concepts seemed to overlap; compartmentalisation was not uniquely related to any of the recovery stages. These findings suggest that recovery from psychosis does not require processes such as compartmentalisation, although these may play a part in explaining processes such as ‘sealing over’ of psychotic symptoms,^45^ a commonly observed clinical phenomenon.

QPR was a significant predictor of STORI. This finding could be seen as a validation of the rating scales. QPR encompasses psychological well-being, quality of life and empowerment, which are generally seen as crucial in recovery from psychosis^43^ and are goals pursued throughout the journey of recovery and achieved in the latest stages of recovery.^39^ In addition, people who have less disturbing psychotic symptoms or very few residual symptoms (who are likely to include the sample recruited) will be able to achieve more advanced recovery goals and place themselves higher on the recovery stages. There may be some who continue to have high levels of residual symptoms but are still able to reach advanced stages of recovery through engagement and therapeutic interventions that enable empowerment.

History of substance misuse and history of trauma were not significant predictors of recovery stages either; however, total PANSS score marginally predicted STORI. This suggests that recovery, at least measured in this way, may be linked to changes in the primary pathology, for example, as part of an antipsychotic treatment response.

Our correlation analyses showed that QPR and STORI were strongly corelated. Notably, PANSS was not correlated with QPR. This potentially suggests that individuals can progress through stages of recovery and demonstrate recovery as evidenced by the QPR, by developing insight, though without a significant decrease in symptoms as assessed by the PANSS. It also implies that as we would expect from clinical experience, dissociation and psychosis are different variables, and that successful treatment of psychosis may not affect dissociative symptoms, but these symptoms may be reasons for different outcomes and recovery in psychosis.

We found that that the QPR was negatively correlated with DES and DCI, whereas STORI showed no association with DES or DCI. This raised the question of how these findings could be explained, as both QPR and STORI are recovery measures and correlated with each other, as well as why recovery process rather than stage of recovery was correlated with dissociation. The finding may have been due to measurement issues related to the DES and DCI, as detailed above. The Spearman's correlations showed that STORI was not correlated with any other variable. This is potentially further evidence that STORI represents a more descriptive measure of recovery, rather than a quantitative measure suitable for use in evaluation of clinical progression. There are concerns that the STORI inventory does not differentiate clearly between the stages of recovery. Its authors have reported^42^ that the instrument does not discriminate sufficiently between the five stages defined by the recovery model that has at its core the concept of recovery as defined by patients. Further research will need to focus on refinement of this instrument to enable it to capture the concepts characteristic of the five recovery stages as defined by the model. We acknowledge that the instruments that we used in this study have been variously effective in their intended roles and hope that this finding will be helpful for researchers designing future research projects.

Perona-Garcelán et al^46^ observed that patients with hallucinations and those who had recovered from them had higher percentages of dissociative experiences than patients with psychosis who had never had hallucinations and the non-clinical group. Their findings show that although participants with hallucinations had more depersonalisation and absorption experiences, those who had recovered from hallucinations continued to be highly absorbed but did not have depersonalisation experiences. Humpston et al^47^ noted that the nature of absorption as a mediating factor could vary depending on whether the individual was already psychotic or was in a pre-psychotic or psychosis-prone stage. Absorption is normal, yet it appears equally in acute illness and after recovery; depersonalisation appears in acute illness but does not seem to appear after recovery. So, we remain curious about how these experiences are related to recovery from psychosis, and we believe that further exploration of how these experiences occur in the recovery process is needed.

Our research was preliminary and restricted to the question of the possible association between dissociation and recovery in psychosis. Future research will need to consider the potential role of more specific biological, social or psychological mechanisms in recovery from psychosis. For the purposes of this study, we included participants with a mixed psychosis diagnosis (rather than more tightly defined diagnostic groups such as schizophrenia), first, because we were interested in exploring the presence of any association between psychosis in general and the three dissociative phenomena, and, secondly, because recruitment was carried out in one NHS organisation. The challenges of recruiting from this group of patients are well known and numerous; to these were added restrictions resulting from the Covid pandemic. We hope that the possibility of replicating this study in a larger group of participants with more tightly defined diagnoses, including consideration of specific diagnoses, will be considered in a future research project. The correlations that we found between recovery as measured by the QPR and dissociative experiences as measured by the DES and DCI suggest that as an individual recovers from psychosis, repeated exposure to situations that require the use of coping mechanisms trains the person to adapt more quickly, without the need to dissociate to deal with stressful situations. This assumption, however, remains to be explored in a future study.

Future research could also analyse specific relevant recovery items that might help us to understand more clearly the relationship between psychosis and dissociation, for example, quality of life and level of functioning.

We believe that our findings are interesting and that some clinical applications may be derived from this work. For example, in research on the treatment of psychosis, it would be of interest to identify response to treatment of dissociative experiences; this could potentially help in the design of a range of pharmacological and psychological interventions appropriate for this complex clinical situation. Our findings suggest that we cannot assume that dissociative experiences occurring as part of a psychotic presentation will respond to standard treatment; other approaches, including psychological interventions and eye movement desensitisation and reprocessing, may be needed.

We share the view of Humpston et al^47^ that there are conceptual overlaps among detachment, compartmentalisation and absorption. This will need to be considered by future research. As the current literature on dissociation is relatively limited and non-homogenous, it may be helpful to go back to consider the central phenomenology of the process (or processes) of dissociation to enable us to improve our understanding and define these important clinical phenomena more accurately. A little-researched concept is that of compartmentalisation; therefore, it seems important to explore the phenomenological construct of compartmentalisation and how this relates to psychosis. Further research is needed to operationalise the complex concept of recovery that would facilitate recovery-orientated research and practice.

In conclusion, these findings should be considered a preliminary attempt to study dissociation in recovery. It would be of interest to replicate the study using dissociation measurement instruments other than the DES and to include a larger population sample, including consideration of specific diagnoses.

## Data Availability

The data that support the findings of this study are available on request from the corresponding author (C.C.). The data are not publicly available for sharing owing to ethical approval requirements.

## Notes

**Claudia Calciu** is a consultant psychiatrist at Gloucestershire Health and Care NHS Foundation Trust, Gloucester, UK; and a doctorand at the Psychology Faculty, School of Psychology and Educational Sciences, ‘Alexandru Ioan Cuza’ University, Iasi, Romania. **Rob Macpherson** is a consultant psychiatrist at Gloucestershire Health and Care NHS Foundation Trust, Gloucester, UK. **Kerry J. Rees** is a senior lecturer in psychology at the University of Gloucestershire, Cheltenham, UK. **Sui Yung Chen** is a CT3 psychiatry trainee at Gloucestershire Health and Care NHS Foundation Trust, Gloucester, UK. **Sarah Ruxton** is a CT3 psychiatry trainee at Gloucestershire Health and Care NHS Foundation Trust, Gloucester, UK. **Rhiannon White** is an FY2 doctor at Gloucestershire Health and Care NHS Foundation Trust, Gloucester, UK. **Mazen Almaskati** is a CT3 psychiatry trainee at Gloucestershire Health and Care NHS Foundation Trust, Gloucester, UK. **Francesca Hill** is an advanced psychiatry trainee at Gloucestershire Health and Care NHS Foundation Trust, Gloucester, UK. **Anca Vasilis-Peter** is a specialty doctor at Gloucestershire Health and Care NHS Foundation Trust, Gloucester, UK. **Sebastian Desando** is a CT3 psychiatry trainee at Gloucestershire Health and Care NHS Foundation Trust, Gloucester, UK. **Oliver Pennell** is an occupational therapist at Gloucestershire Health and Care NHS Foundation Trust, Gloucester, UK. **Carolyn Nasubuga** is a CT1 psychiatry trainee at Gloucestershire Health and Care NHS Foundation Trust, Gloucester, UK. **Jackie Webb** is Library Services Manager at Gloucestershire Health and Care NHS Foundation Trust, Gloucester, UK. **Mark Walker** is Head of Research and Development, Gloucestershire Health and Care NHS Foundation Trust, Gloucester, UK. **Camelia Soponaru** is a professor at the Psychology Faculty, School of Psychology and Educational Sciences, ‘Alexandru Ioan Cuza’ University, Iasi, Romania.
